# Mesenchymal Stem Cell-Derived Exosomal miRNAs in Skin Repair and Rejuvenation

**DOI:** 10.3390/genes17040450

**Published:** 2026-04-13

**Authors:** Jijun Hao

**Affiliations:** Department of Foundational Sciences, College of Veterinary Medicine, Western University of Health Sciences, 309 E 2nd Street, Pomona, CA 91766, USA; jhao@westernu.edu; Tel.: +1-(909)-469-8686; Fax: +1-(909)-469-5635

**Keywords:** mesenchymal stem cells, miRNAs, exosomes, skin aging, wound healing

## Abstract

Skin aging and wound healing are the result of intricate and interconnected processes involving chronic inflammation, oxidative stress, cellular senescence and extracellular matrix degradation. Mesenchymal stem cell (MSC)-derived exosomes are rich in bioactive components, particularly microRNAs (miRNAs), which play a crucial role in regulating gene expression and key signaling pathways critical for maintaining skin homeostasis. This article reviews the current evidence regarding the roles of MSC-derived exosomal miRNAs (MSC-Exo-miRNAs) in cutaneous repair and rejuvenation. Specific exosomal miRNAs are analyzed for their ability to modulate inflammatory responses, promote fibroblast proliferation and collagen synthesis, enhance angiogenesis, and facilitate keratinocyte migration and re-epithelialization. Their roles in regulating key signaling pathways are discussed in the context of skin regeneration and aging, including nuclear factor-κB (NF-κB), PI3K/Akt, TGF-β/Smad, Wnt/β-catenin, and nuclear factor erythroid 2-related factor 2 (Nrf2). Additionally, emerging engineering strategies aimed at optimizing miRNA cargo loading, improving delivery efficiency, and advancing clinical translation are highlighted. Overall, MSC-Exo-miRNAs represent a promising cell-free therapeutic strategy for skin repair and rejuvenation; however, further mechanistic investigations and rigorous clinical studies are necessary to fully realize their translational potential.

## 1. Introduction

Skin aging and impaired wound healing are interrelated processes that share significant underlying molecular and cellular mechanisms. The skin functions as the body’s primary protective barrier and plays crucial roles in immune defense, thermoregulation, and tissue repair. As aging progresses, both intrinsic and extrinsic factors contribute to the skin’s gradual structural and functional decline, including epidermal thinning, reduced dermal collagen, impaired vascularization and slower regenerative responses [1,2]. These age-related changes not only contribute to the visible signs of skin aging but also weaken the skin’s ability to repair itself. Consequently, chronic non-healing wounds, such as diabetic ulcers, venous leg ulcers and pressure injuries, have become increasingly prevalent, leading to significant morbidity and imposing a substantial economic burden on healthcare systems worldwide [3].

At the molecular level, skin aging and impaired wound healing arise from several interconnected mechanisms, including chronic low-grade inflammation, oxidative stress driven by reactive oxygen species (ROS), cellular senescence and degradation of the extracellular matrix (ECM). Persistent activation of nuclear factor-κB (NF-κB) signaling increases the production of pro-inflammatory cytokines [4], while ROS promote mitochondrial dysfunction and accelerate collagen degradation [5]. Additionally, senescent cells release inflammatory mediators through the senescence-associated secretory phenotype (SASP), further disrupting tissue homeostasis [6]. Elevated matrix metalloproteinase (MMP) activity also contributes to the breakdown of collagen types I and III, which weakens dermal structure and impairs normal wound remodeling [7].

Mesenchymal stem cell (MSC)-based therapies have demonstrated considerable promise in promoting tissue regeneration and wound repair. However, increasing evidence suggests that many of the therapeutic benefits of MSCs arise primarily from paracrine mechanisms, particularly through the release of extracellular vesicles such as exosomes [8]. Exosomes are small extracellular vesicles (approximately 30–150 nm in diameter) generated through the endosomal pathway and play a significant role in intercellular communication [9]. MSC-derived exosomes carry a variety of bioactive molecules including proteins, lipids and nucleic acids, particularly microRNAs (miRNAs) [10]. These MSC-derived exosomal miRNAs (MSC-Exo-miRNAs) modulate a broad range of processes implicated in inflammation, angiogenesis, fibroblast proliferation, collagen synthesis, keratinocyte migration, and cellular senescence [9,11]. Despite these promising findings, significant challenges remain in standardizing exosome isolation protocols, optimizing miRNA cargo enrichment, improving targeted delivery and advancing clinical translation. This article reviews current studies of MSC-Exo-miRNAs in regulating inflammation, ECM remodeling, angiogenesis, keratinocyte migration and cellular senescence in the context of skin repair and aging. It also discusses emerging engineering strategies to enhance their therapeutic efficacy and key translational considerations for future clinical applications.

## 2. MSC-Exo-miRNAs in Regulation of Skin Inflammation

**Figure 1:**
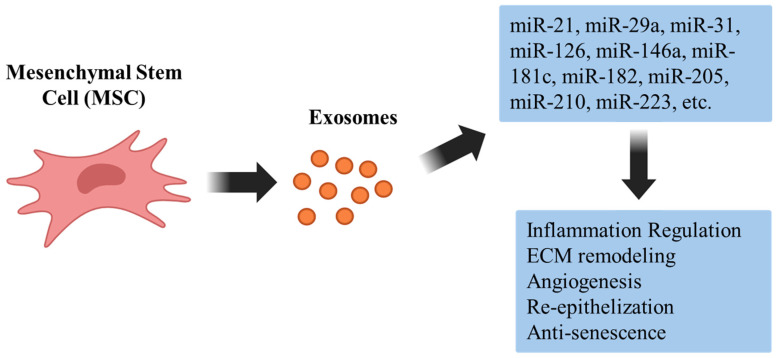
Schematic illustration of MSC-derived exosomes and their miRNA cargo in skin regeneration. MSC-secreted exosomes contain regulatory miRNAs (e.g., miR-21, miR-29a, miR-31, miR-126, miR-146a, miR-181c, miR-182, miR-205, miR-210, and miR-223) that influence key biological processes, including inflammation regulation, extracellular matrix remodeling, angiogenesis, keratinocyte re-epithelialization, and anti-senescence signaling.

Chronic inflammation is central to both skin aging and impaired wound healing. By modulating cytokine expression and immune cell behavior at the post-transcriptional level, MSC-Exo-miRNAs have emerged as important regulators of these cutaneous inflammatory responses [12]. At the cytokine level, several MSC-Exo-miRNAs directly target key pro-inflammatory mediators (Figure 1, Table 1). Among these, miR-146a suppresses inflammatory signaling via the NF-κB pathway, thereby reducing the production of pro-inflammatory cytokines such as TNF-α and IL-6 [13,14]. Similarly, miR-21 diminishes pro-inflammatory cytokine expression while promoting tissue repair in cutaneous wound models [15]. Other miRNAs, including miR-223 and miR-181c, inhibit cytokine secretion by modulating Toll-like receptor–associated pathways and their downstream transcription factors [16,17,18]. Collectively, these miRNAs fine-tune the inflammatory microenvironment of aged or injured skin through coordinated post-transcriptional regulation.

Macrophage polarization represents another crucial mechanism through which MSC-Exo-miRNAs influence the wound healing response. Effective tissue repair necessitates a well-coordinated transition from pro-inflammatory M1 macrophages to anti-inflammatory and pro-regenerative M2 macrophages [19]. Several miRNAs carried by MSC-derived exosomes contribute to this process. For instance, miR-223 and miR-182 have been shown to promote the M1-to-M2 shift, enhancing anti-inflammatory gene expression and accelerating wound closure [20,21]. miR-146a further supports this transition by suppressing NF-κB–dependent transcription, thereby helping to resolve excessive inflammation and creating a tissue environment more conducive to regeneration [22].

These MSC-Exo-miRNAs-mediated immunomodulatory effects are closely linked to the NF-κB signaling pathway, a central regulator of cutaneous inflammation and aging. Chronic activation of NF-κB promotes sustained expression of pro-inflammatory cytokines, chemokines, and matrix-degrading enzymes that contribute to persistent inflammation in aged or injured skin [23]. Suppression of NF-κB activity by MSC-Exo-miRNAs thus helps interrupt this inflammatory cycle and can also reduce the SASP, a key driver of inflammaging and impaired wound repair [8]. In summary, the literature suggests that MSC-Exo-miRNAs act as versatile regulators of cutaneous inflammation. By modulating cytokine networks, guiding macrophage phenotype transitions, and influencing central transcriptional pathways, they help create a molecular environment that supports both efficient skin regeneration and healthy aging.

## 3. MSC-Exo-miRNAs in Fibroblast Proliferation, Collagen Synthesis and ECM Remodeling

Dermal fibroblasts are the primary effector cells responsible for maintaining ECM homeostasis and the structural integrity of the skin, playing crucial roles in both skin repair and cutaneous aging. During wound healing, fibroblasts proliferate, migrate into the wound bed, differentiate into myofibroblasts and deposit collagen and other matrix components to restore tissue architecture. In aged skin, fibroblasts show reduced proliferative capacity, diminished collagen synthesis, elevated matrix metalloproteinase (MMP) activity and SASP, which collectively drive dermal thinning, loss of elasticity and impaired barrier function [24,25]. Oxidative stress and chronic inflammation further exacerbate these changes, creating a microenvironment that accelerates skin aging while compromising wound repair capacity. MSC-Exo-miRNAs have demonstrated critical roles in regulating fibroblast behavior and ECM remodeling in both contexts, offering a unified therapeutic approach to restoring dermal homeostasis [21] (Table 1).

Among the most studied miRNAs in this context, miR-21 promotes fibroblast proliferation and migration by activating the PI3K/Akt signaling pathway and suppressing phosphatase and tensin homolog (PTEN), a negative regulator of cell survival [26,27]. The resulting Akt activation stimulates fibroblast growth, cytoskeletal reorganization and resistance to apoptosis, which collectively accelerate wound closure. Beyond proliferation, miR-21 also engages TGF-β signaling, a central driver of collagen synthesis and myofibroblast differentiation [28]. When appropriately modulated, TGF-β/Smad activation supports collagen deposition during repair; however, when dysregulated, it tips the balance toward pathological fibrosis. Exosomal delivery of miR-21 offers a context-sensitive mechanism for navigating this fine balance [28]. miR-29 represents another important regulator of ECM homeostasis, and it modulates collagen synthesis by targeting multiple collagen-encoding genes (COL1A1, COL3A1) and fibrosis-related factors [29]. In aged or damaged skin, reduced miR-29 expression leads to excessive collagen accumulation and dysregulated matrix turnover. Restoring miR-29 levels through exosomal delivery recalibrates this balance by suppressing pathological collagen overproduction while preserving the structural collagen synthesis necessary for effective repair and tissue restoration [30,31]. Beyond miR-21 and the miR-29 family, exosomal miR-135a derived from human amnion mesenchymal stem cells has been shown to promote cutaneous wound healing and enhance fibroblast migration by directly inhibiting large tumor suppressor kinase 2 (LATS2), thereby contributing to extracellular matrix remodeling and tissue regeneration [32].

Moreover, these miRNAs regulate extracellular matrix remodeling by controlling MMPs and their endogenous inhibitors (TIMPs). Elevated MMP-1 and MMP-9 activity is a hallmark of both photoaged and chronically inflamed skin, driving collagen fragmentation and progressive dermal deterioration [24,33]. miR-21, acting via PI3K/Akt, modulates MMP-9 expression and fine-tunes TIMP-1 activity during wound repair, while miR-29a reduces pathological MMP dysregulation by suppressing the TGF-β2/Smad3 axis [34]. Together, these miRNAs help shift the MMP/TIMP balance toward matrix preservation, promoting more organized collagen architecture and improved biomechanical outcomes in regenerated skin.

## 4. MSC-Exo-miRNAs in Angiogenesis and Vascular Remodeling

Vascularization is essential for effective wound healing as it ensures that the regenerating tissue receives adequate oxygen and nutrients throughout the healing process. Impaired neovascularization is a common characteristic of chronic wounds and aged skin, and is particularly evident in diabetic and ischemic conditions where endothelial dysfunction further impairs tissue perfusion [35] (Table 1). MSC-Exo-miR-126 enhances endothelial proliferation and migration by promoting angiogenic responses through the vascular endothelial growth factor (VEGF) signaling pathway via suppression of its negative regulators such as SPRED1 and PIK3R2 [26,36]. As a key regulator of angiogenesis, VEGF activation facilitates endothelial survival, tube formation, and vascular maturation and MSC-derived exosomes enriched with miR-126 have demonstrated increased capillary density and improved tissue perfusion in preclinical wound models [36].

Furthermore, in diabetic and ischemic wound models where hyperglycemia-induced endothelial dysfunction and oxidative stress significantly hinder angiogenic responses, MSC-Exo-miRNAs have exhibited notable restorative effects. Exosomal miR-210, for example, enhances endothelial migration and tube formation under hypoxic conditions by modulating hypoxia-inducible factor-1 alpha (HIF-1α)–dependent pathways [37]. More broadly, exosome treatment in diabetic wound models has been shown to increase microvascular density, accelerate granulation tissue formation and reduce overall healing time [38]. These findings suggest that MSC-Exo-miRNAs not only support angiogenesis under normal physiological conditions but can also restore vascular function in environments where it is most severely compromised. Collectively, through the enhancement of VEGF signaling and modulation of hypoxia-responsive cascades, exosomal miRNAs drive vascular remodeling and restore perfusion in injured and aged skin, making their pro-angiogenic capacity a particularly compelling therapeutic attribute for chronic wounds and age-related vascular decline.

## 5. MSC-Exo-miRNAs in Keratinocyte Migration and Re-Epithelialization

Re-epithelialization constitutes the final and functionally critical phase of wound healing, necessitating the coordinated proliferation, migration and differentiation of keratinocytes to reestablish an intact epidermal barrier. In aged or chronically inflamed skin, this regenerative capacity is significantly reduced, resulting in delayed barrier restoration and heightened vulnerability to infection and further injury [39]. MSC-Exo-miRNAs can facilitate epidermal repair through multiple keratinocyte signaling networks (Table 1). At the cellular level, miR-21 and miR-31 expedite wound closure by activating the PI3K/Akt and extracellular signal-regulated kinase signaling pathways, thereby enhancing cytoskeletal reorganization and inhibiting apoptosis in migrating keratinocytes [40,41]. miR-205 contributes complementarily by maintaining epithelial integrity and facilitating keratinocyte migration through the modulation of epithelial junction proteins [42]. Collectively, these miRNAs drive the leading edge of re-epithelialization while preserving the structural cohesion of the advancing epithelial sheet. Effective barrier restoration extends beyond cell migration and equally depends on proper keratinocyte differentiation and tight junction assembly. Exosomal miRNAs support this process by regulating key barrier-associated proteins (filaggrin, involucrin, and claudins) and modulating the transcriptional programs that govern epidermal maturation [43]. This regulatory layer ensures that the newly formed epithelium is not only structurally continuous but also functionally competent.

The Wnt/β-catenin signaling pathway further contributes to re-epithelialization by promoting keratinocyte proliferation and activating hair follicle–associated stem cells during wound repair. MSC-derived exosomes have been shown to enhance Wnt/β-catenin signaling in cutaneous cells, thereby stimulating regenerative responses and accelerating epidermal repair [33]. Notably, exosomal delivery of miR-21-5p has been demonstrated to promote keratinocyte proliferation and migration via activation of Wnt/β-catenin signaling, thereby accelerating re-epithelialization and wound closure in diabetic wound models [44]. Interactions between Wnt, TGF-β, and PI3K/Akt signaling pathways further integrate epidermal regeneration with dermal remodeling processes, coordinating surface repair with deeper tissue reconstruction. Additionally, exosomal miRNAs have been reported to influence other developmental pathways involved in epidermal differentiation and stem cell maintenance, including Notch and Hedgehog signaling [45]. In summary, the capacity of MSC-Exo-miRNAs to enhance keratinocyte proliferation, migration, differentiation, and barrier reconstitution complements their pro-angiogenic and fibroblast-stimulatory roles, reinforcing the multi-layered regenerative potential of MSC-derived exosomes in cutaneous repair and aging.

## 6. MSC-Exo-miRNAs and Cellular Senescence in Skin Aging

Cellular senescence is a significant contributor to skin aging and impaired wound healing. The accumulation of senescent cells in the aged dermis and epidermis establishes a tissue environment characterized by the SASP [46]. This environment perpetuates a cycle of inflammation, ECM degradation and reduced regenerative capacity, which contribute to the most recognizable features of aged skin [47]. MSC-Exo-miRNAs have garnered increasing interest as modulators of these senescence-associated processes, offering a biologically grounded approach to dermal rejuvenation (Table 1).

The molecular mechanisms driving senescence are centered on two interconnected tumor suppressor pathways: p53/p21 and p16INK4a/retinoblastoma protein (Rb). Upon reaching a critical threshold of DNA damage or oxidative stress, p53 is activated, leading to the transcription of p21, which halts the cell cycle. Concurrently, p16INK4a independently induces senescence by inhibiting cyclin-dependent kinases [48,49]. MSC-Exo-miR-146a has been reported to suppress p53-associated stress responses and reduce the inflammatory signaling associated with senescence [50], while the exosomal miRNAs targeting p16INK4a can decrease the burden of senescent cells and partially restore proliferative activity in aged tissues [25]. Although these effects require further characterization in human skin, they suggest a potential to delay or mitigate senescence-driven dysfunction. Oxidative stress serves as a critical upstream trigger in this senescence cascade. With advancing age and cumulative environmental exposure, ROS accumulate and damage mitochondria and DNA, thereby promoting both p53-dependent arrest and NF-κB–driven inflammaging [5,46]. Exosomal miRNAs counteract this by enhancing cellular antioxidant defenses. Notably, miR-29a-3p activates the nuclear factor erythroid 2–related factor 2 (Nrf2) antioxidant pathway, which induces cytoprotective enzymes such as heme oxygenase-1 and NAD(P)H quinone oxidoreductase 1 [51]. MSC-derived exosomes broadly activate Nrf2 signaling and reduce intracellular ROS levels, leading to improved fibroblast viability and collagen synthesis under oxidative stress conditions [52]. By suppressing SASP-associated inflammation, rebalancing redox signaling and promoting a more regenerative phenotype in senescent fibroblasts, MSC-Exo-miRNAs offer a comprehensive approach to enhancing collagen synthesis, dermal thickness and skin elasticity [53].

## 7. Engineering Strategies to Enhance MSC-Exo-miRNAs Therapeutic Efficacy

While MSC-Exo-miRNAs exhibit significant therapeutic potential, their successful application in clinical settings necessitates strategic enhancements in cargo enrichment, targeting specificity and delivery efficiency. Recent advancements in cellular engineering and biomaterial science have yielded several practical approaches to address these challenges. A widely investigated strategy involves preconditioning MSCs prior to exosome collection. Culturing MSCs under hypoxic conditions enhances the secretion of pro-angiogenic miRNAs such as miR-126 and miR-210 through the activation of HIF-1α [54,55]. The exosomes produced under these conditions demonstrate enriched angiogenic cargo and have shown improved vascularization and wound repair outcomes in vivo [56]. Cytokine-based preconditioning with inflammatory mediators, such as TNF-α or IFN-γ, offers a complementary approach: preconditioning MSCs with TNF-α significantly increases exosome yield, enriches miRNA cargo, including miR-146a-5p, and modulates the exosomal miRNA profile to enhance immunomodulatory properties [57,58]. Collectively, these strategies enable the tailoring of exosome therapeutic cargo to specific pathological contexts.

Genetic modification of MSCs provides a more direct method for cargo enrichment. Overexpressing selected miRNAs through viral or plasmid-based transfection results in their preferential packaging into secreted exosomes, thereby amplifying specific therapeutic signals [59]. For instance, MSCs engineered to overexpress miR-146a produce exosomes with significantly enhanced anti-inflammatory and pro-regenerative properties [13]. This approach retains the advantages of cell-free delivery while allowing precise amplification of desired therapeutic pathways. Increasing attention has recently been directed to ensuring that exosomes effectively reach and accumulate at the intended tissue site, which remains a significant challenge. Surface engineering approaches offer a promising solution by conjugating targeting peptides to the exosomal membrane or by genetically fusing homing ligands to membrane-anchored proteins such as lysosome-associated membrane glycoprotein 2b [60,61]. It is possible to direct exosome distribution toward injured skin or ischemic tissues, thereby improving on-target delivery and reducing systemic dilution and off-target accumulation that limit the efficacy of unmodified preparations [21,62]. These modifications represent a significant advancement toward tissue-selective exosome delivery in clinical wound care.

Biomaterial-based platforms address a different but equally important challenge: maintaining sufficient exosome concentration and stability at the wound site. Incorporating exosomes into hydrogels, collagen scaffolds or chitosan-based matrices enables sustained and controlled release while protecting cargo from rapid degradation [12,63]. Hydrogels are particularly well-suited to this application, providing a moist wound microenvironment that supports healing while facilitating gradual exosome diffusion. Scaffold-based systems additionally lend themselves to integration with existing wound dressings and tissue-engineering constructs, broadening their clinical applicability. Across chronic wound models, these platforms have consistently improved local exosome concentration, prolonged therapeutic exposure and enhanced regenerative outcomes [64]. In summary, preconditioning, genetic engineering, surface modification and biomaterial-based delivery constitute a complementary toolkit for overcoming the current translational limitations of MSC-Exo-miRNA therapy. Continued refinement of these strategies will be critical to unlocking their full clinical potential in skin repair and rejuvenation.

## 8. Current Challenges and Translational Considerations

Despite compelling preclinical evidence, the integration of MSC-Exo-miRNA therapy into standard clinical practice remains a significant challenge, with obstacles encompassing scientific, technical and regulatory domains. Prior to addressing these translational barriers, it is essential to recognize that the evidence base is not uniformly supportive, necessitating a candid assessment of its limitations to accurately ascertain the current status of the field.

The therapeutic potential of MSC-Exo-miRNAs is highly contingent upon the specific context, with several findings that initially appear promising becoming more complex upon detailed examination. For example, signaling pathways that promote wound closure may, under certain biological conditions, also contribute to aberrant fibroproliferative responses or tumorigenesis. Similarly, exosomal miR-146a can suppress NF-κB–mediated inflammatory signaling in macrophages, yet the downstream cytokine responses vary considerably depending on the inflammatory stimulus, cell type and exosome dosage [65]. Members of the miR-29 family illustrate a similar biological tension. Although miR-29 is widely regarded as anti-fibrotic due to its ability to suppress collagen gene expression, excessive or premature inhibition of collagen synthesis may impair early wound repair, during which a transient increase in extracellular matrix deposition is required for proper tissue regeneration [66]. These observations reflect genuine biological complexity rather than minor inconsistencies and highlight the limitations of relying on single-model experimental systems.

MSC heterogeneity represents a significant yet insufficiently characterized source of variability in cross-study analyses of MSC-Exo-miRNAs. Bone marrow-derived (BM-MSCs), adipose-derived (AD-MSCs), and umbilical cord-derived MSCs (UC-MSCs) produce exosomes with distinct miRNA profiles, reflecting differences in their underlying transcriptional programs [67]. For instance, exosomes derived from AD-MSCs are typically enriched with miRNAs associated with angiogenesis and lipid metabolism, whereas those from UC-MSCs reflect the immunotolerant characteristics of perinatal tissue through a higher concentration of immunomodulatory miRNAs [68]. BM-MSC exosomes are more frequently linked to matrix remodeling contexts. These intrinsic differences are further exacerbated by factors such as donor age, metabolic status and passage number during cell expansion, all of which progressively diminish miRNA cargo quality and regenerative potential [69]. As a result, inconsistencies across studies likely reflect differences in MSC-Exo-miRNA composition rather than intrinsic miRNA effects alone.

A particularly pressing practical issue is the absence of standardized protocols for exosome isolation and characterization. Preparations vary significantly depending on whether ultracentrifugation, size-exclusion chromatography, precipitation-based techniques or microfluidic platforms are employed, and these methodological differences directly impact particle purity, yield and cargo composition [70]. The International Society for Extracellular Vesicles has addressed this issue by establishing minimum reporting criteria under MISEV2023, which include size distribution profiling, detection of canonical surface markers (CD9, CD63 and CD81) and functional validation [71]. Nevertheless, inconsistent adherence to these guidelines continues to limit the reproducibility and cross-study comparability of MSC-Exo-miRNA findings.

Another major challenge lies in the delivery efficiency and biodistribution of MSC-Exo-miRNAs. Therapeutic outcomes are influenced by exosome dose, administration frequency and delivery route. Systemically administered exosomes often accumulate in the liver, spleen, and lungs, limiting effective delivery of miRNA cargo to target tissues such as wounds [21]. Local delivery strategies, including hydrogel-based systems, improve retention and sustained release of MSC-Exo-miRNAs at injury sites, but robust pharmacokinetic and pharmacodynamic data remain limited [35,72].

Although safety profiles of MSC-Exo-miRNAs are generally favorable, they still require careful evaluation. Exosomes are considerably less immunogenic than live cell therapies; however, the extensive regulatory reach of their miRNA cargo poses a theoretical risk of unintended off-target gene modulation [59]. Prolonged activation of pathways such as PI3K/Akt or TGF-β, while beneficial in acute wound repair, could potentially contribute to fibrosis or neoplastic transformation under extended exposure [35]. Systematic long-term assessments of immunogenicity, proliferative risk and pro-fibrotic potential are essential before clinical adoption can proceed with confidence. Scaling production to meet clinical demand presents its own challenges. The regulatory classification of exosome-based therapies further complicates the path to market, as different jurisdictions categorize these products variously as biological agents, advanced medicinal therapies or drug-delivery systems, each with distinct and not always compatible approval requirements [73]. The lack of harmonized international standards risks delaying commercialization and restricting patient access. Ultimately, while early-phase clinical trials have reported encouraging safety profiles, large randomized controlled studies are required to establish efficacy and define optimal therapeutic parameters.

## 9. Conclusions

The strategy for skin repair and rejuvenation is significantly advanced by the application of MSC-Exo-miRNAs, as evidenced by their ability to coordinate a wide array of regenerative processes. These processes include the suppression of inflammation, stimulation of fibroblast proliferation and collagen synthesis, promotion of angiogenesis, facilitation of keratinocyte migration and attenuation of cellular senescence. The MSC-Exo-miRNAs’ capacity to engage multiple interconnected pathways, such as NF-κB, PI3K/Akt, TGF-β/Smad, Wnt/β-catenin and Nrf2, provides them with a multi-target regulatory capability that is exceptionally well-suited to the complex biology of aged and wounded skin.

However, the path to clinical translation remains challenging. Standardization of isolation protocols, GMP-compliant manufacturing at scale, and unresolved questions regarding dosing, biodistribution and long-term safety require systematic investigation. Future efforts must focus on deepening the mechanistic understanding of miRNA–gene networks in aging and wound microenvironments, refining cargo engineering strategies and developing biomaterial delivery platforms capable of sustained local release. Crucially, well-designed randomized controlled trials in patients with chronic wounds and age-related dermal conditions are essential to transition from biological promise to clinical evidence. MSC-Exo-miRNAs represent a scientifically grounded and potentially transformative approach to cutaneous regeneration. With rigorous translational development, they hold the potential not only to enhance existing treatments but also to fundamentally alter the understanding and management of skin aging and impaired wound healing.

## Figures and Tables

**Table 1:** Mechanistic roles of MSC-derived exosomal miRNAs in skin repair and rejuvenation, including their molecular targets, signaling pathways, and functional effects.

Biological Process	Molecular Target/Signaling Pathway	Key MSC-Exo-miRNAs	Functional Effect
Inflammation Regulation	NF-κB signaling	miR-146a, miR-181c	Suppresses NF-κB activity and reduces pro-inflammatory cytokines (e.g., TNF-α, IL-6)
	Macrophage polarization	miR-223, miR-182	Promotes macrophage polarization from M1 (pro-inflammatory) to M2 (anti-inflammatory) phenotype
ECM Remodeling	Fibroblast proliferation (PI3K/Akt pathway)	miR-21	Suppresses PTEN, leading to activation of PI3K/Akt signaling and enhanced fibroblast proliferation and survival
	Collagen synthesis (TGF-β/Smad pathway)	miR-21, miR-29a	miR-21 promotes TGF-β/Smad signaling; miR-29a suppresses COL1A1/COL3A1 to regulate collagen balance
	Matrix remodeling (MMP/TIMP balance)	miR-21, miR-29a	Regulates MMP-9 and TIMP activity to maintain extracellular matrix integrity
	Fibroblast migration (LATS2/Hippo pathway)	miR-135a	Inhibits LATS2, promoting fibroblast migration and contributing to tissue remodeling
Angiogenesis	VEGF signaling (PI3K/Akt pathway)	miR-126	Suppresses SPRED1 and PIK3R2, enhancing endothelial proliferation, migration, and tube formation
	Hypoxia response (HIF-1α pathway)	miR-210	Enhances endothelial migration and angiogenesis under hypoxic conditions
Re-epithelialization	Keratinocyte migration (PI3K/Akt, ERK pathways)	miR-21, miR-31, miR-205	Promotes keratinocyte proliferation, migration, and epithelial integrity
	Wnt/β-catenin signaling	miR-21-5p	Promotes Wnt/β-catenin signaling to enhance keratinocyte proliferation and wound closure
Anti-senescence	NF-κB–associated inflammatory signaling	miR-146a	Attenuates inflammation-associated senescence pathways
	Oxidative stress (Nrf2 pathway)	miR-29a-3p	Activates Nrf2 signaling, enhancing antioxidant defense and reducing ROS

## Data Availability

No new data were created or analyzed in this study. Data sharing is not applicable to this article.
